# Acute impact of retrograde shear rate on brachial and superficial femoral artery flow‐mediated dilation in humans

**DOI:** 10.1002/phy2.193

**Published:** 2014-01-06

**Authors:** Tim H. A. Schreuder, Daniel J. Green, Maria T. E. Hopman, Dick H. J. Thijssen

**Affiliations:** 1 Department of Physiology, Radboud University Nijmegen Medical Centre, Nijmegen, The Netherlands; 2 Research Institute for Sport and Exercise Science, Liverpool John Moores University, Liverpool, United Kingdom; 3 School of Sport Science, Exercise and Health, The University of Western Australia, Crawley, Western Australia, Australia

**Keywords:** Atherosclerosis, echo doppler, endothelial function, retrograde shear stress, shear stress pattern

## Abstract

Retrograde shear rate (SR) in the brachial artery (BA) is associated with endothelial dysfunction; a precursor to atherosclerosis. The BA does not typically manifest clinical atherosclerosis, whereas the superficial femoral artery (SFA) is more prone to developing plaque. Examine whether the impact of incremental levels of retrograde SR differs between atherosclerosis‐prone (i.e., SFA) and ‐resistant vessels (i.e., BA) in healthy men. Thirteen healthy young men reported three times to the laboratory. We examined BA flow‐mediated dilation (FMD) before and after 30‐min exposure to cuff inflation around the forearm at 0, 30, and 60 mmHg, to manipulate retrograde SR. Subsequently, the 30‐min intervention was repeated in the SFA, using the same cuff pressure as in the forearm. Order of testing (vessel and intervention) was randomized among subjects. We found a dose‐dependent increase in retrograde SR with 30 and 60 mmHg cuff inflation, which was present in both the BA and SFA (all *P* < 0.05). BA and SFA FMD decreased after the 30‐min intervention (“time”: *P* = 0.012), and this was dependent on cuff pressure (“cuff × time”: *P* = 0.024). A significant decrease in FMD was observed after 60 mmHg only and this change was similarly present in both arteries (“time × artery”: *P* = 0.227). Moreover, the BA and SFA demonstrate a similar relationship between changes in retrograde SR and FMD (*r* = 0.498 and 0.475, respectively). Our study demonstrates that acute exposure to an increase in retrograde shear leads to comparable decreases in FMD in atherosclerotic‐prone and ‐resistant conduit arteries in humans.

## Abstract (precis)

We examined whether the impact of incremental levels of retrograde shear rate differs between atherosclerosis‐prone (i.e., superficial femoral artery) and ‐resistant vessels (i.e., brachial artery) in healthy men. Our study demonstrates that acute exposure to an increase in retrograde shear leads to comparable decreases in flow‐mediated dilation in atherosclerotic‐prone and ‐resistant conduit arteries in humans.

## Introduction

Shear stress, that is, the frictional force of blood on the arterial wall, represents an important stimulus for arteries to adapt (Niebauer and Cooke 1996); Thijssen et al. 2009); Tinken et al. 2009). Changes in shear stress directly influence the endothelium of arteries, which plays a crucial role in the regulation of blood flow and maintenance of the quality of blood vessels (Davignon and Ganz 2004). Across the cardiac cycle, shear stress demonstrates a typical pattern, flowing toward the periphery during systole (antegrade shear), and back to the heart during diastole (retrograde shear). Previous animal studies demonstrated that antegrade shear stress is associated with positive, antiatherogenic effects on the endothelium (Chappell et al. 1998); Hsiai et al. 2003); Newcomer et al. 2008); Tinken et al. 2009), 2010); Wang et al. 2013), whereas retrograde shear stress is associated with proatherogenic effects on the endothelium (Widlansky et al. 2003); Thijssen et al. 2009); Newcomer et al. 2011). In a previous study in humans, we manipulated the magnitude of retrograde shear rate and reported an inverse and dose‐dependent relationship between retrograde shear rate and brachial artery endothelial function (Thijssen et al. 2009). However, the impact of retrograde shear rate on endothelial function may differ between different arteries.

The brachial artery is not commonly associated with atherosclerosis‐related complications (Dalager et al. 2007), which raises the question of whether the impact of retrograde shear rate on endothelial function can be extrapolated to other vascular beds, such as the atherosclerotic‐prone arteries of the lower limbs. Previous studies have examined dilatory responses to ischemia‐induced increases in shear (Parker et al. 2006); Nishiyama et al. 2007) and intraarterial infusion of vasoactive substances (Newcomer et al. 2004) in upper and lower limb arteries of young subjects and found more pronounced dilator responses in the upper limbs. These observations suggest differences in vascular responsiveness between upper and lower limbs in healthy, asymptomatic subjects. To date, no study has compared the impact of exposure of retrograde shear on conduit artery endothelial function between lower and upper limbs.

Therefore, the principle aim of this study was to compare the immediate (i.e., 30 min) impact of incremental steps of retrograde shear rate on brachial artery and superficial femoral artery endothelial function in healthy young men. We hypothesized that exposure to incremental levels of retrograde shear rate would result in a smaller decrease in an upper limb, atherosclerosis‐resistant conduit artery compared to a proatherosclerotic, lower limb artery. For this purpose, we examined flow‐mediated dilation (FMD, a surrogate measure of endothelial function) before and after 30‐min manipulation of retrograde shear rate by cuff inflation around the forearm and thigh.

## Methods

### Subjects

Thirteen healthy, recreationally active men (24 ± 3 years, body mass index [BMI]: 22.8 ± 2.7 kg/m^2^) were rec‐ruited from the community. The term recreationally active was defined as 1–7 h of exercise training per week. No subject reported having been diagnosed with cardiovascular disease or risk factors such as hypercholesterolemia or hypertension. Subjects who were on medication influencing the cardiovascular system were excluded. The study procedures were approved by the Ethics Committee of Radboud University Nijmegen Medical Centre, adhered to the Declaration of Helsinki, and all subjects gave prior written consent.

### Experimental design

Each subject reported three times to the laboratory. On each day, we examined the impact of a 30‐min intervention on the brachial and femoral artery endothelial function. Measurements in the upper and lower limb artery were performed consecutively, while the cuff pressure was randomized between days and between subjects. All measurements are done under standardized conditions and unilaterally (i.e., right side). Endothelial function was examined using the FMD, which involves an ischemic stimulus induced by distal cuff inflation to suprasystolic level for 5 min. Brachial and superficial femoral artery FMD was performed before and immediately after each 30‐min intervention, which consisted of inflating an occlusion cuff (placed around the forearm or thigh) to 0, 30, or 60 mmHg (randomized between the 3 days).

### Experimental procedures

Vascular function assessments were conducted in a quiet, temperature‐controlled environment and according to recent expert consensus guidelines (Thijssen et al. 2011). Repeated laboratory visits were conducted at the same time of day to control for diurnal variation. Before each test, subjects were instructed to fast for at least 6 h, abstain from alcohol and caffeine for 18 h, and avoid any exercise for 24 h.

#### Flow‐mediated endothelium‐dependent vasodilator function (FMD%)

Before and after the 30‐min intervention, we assessed the FMD; that is, an endothelium‐dependent, partly nitric oxide‐mediated dilation (Mullen et al. 2001); Green et al. 2005); Kooijman et al. 2008). First, subjects rested in the supine position for a period of at least 15 min to facilitate baseline assessment of heart rate and blood flow. Heart rate, systolic, diastolic, and mean arterial pressure were determined from a manual sphygmomanometer by an experienced researcher, measured twice in the left brachial artery after a resting period in the supine position of at least 5 min. To examine brachial artery FMD, the right arm was extended and positioned at an angle of ~80^°^ from the torso. A rapid inflation and deflation of pneumatic cuff (D.E. Hokanson, Bellevue, WA) was positioned on the forearm of the imaged arm immediately distal to the olecranon process to provide a stimulus to forearm ischemia (Corretti et al. 2002). A 10‐MHz multifrequency linear array probe attached to a high‐resolution ultrasound machine (T3000; Terason, Burlington, MA) was used to image the brachial arteries in the distal one‐third of the upper arm. When an optimal image was obtained, the probe was held stable and the ultrasound parameters were set to optimize the longitudinal, B‐mode images of lumen–arterial wall interface. Continuous Doppler velocity assessment was simultaneously obtained using the ultrasound machine, and was collected using the lowest possible insonation angle (always <60^°^), which did not vary during each study. Baseline images were recorded for 1 min. The forearm cuff was then inflated (>200 mmHg) for 5 min. Diameter and blood flow recordings resumed 30 sec prior to cuff deflation and continued for 3 min thereafter.

After performance of the postintervention measurement of the brachial artery FMD, we repeated this procedure for the superficial femoral artery. Subjects rested supine with the lower leg slightly elevated, while resting on ~15‐cm‐thick foam. The rapid inflation/deflation pneumatic cuff was positioned ~15 cm below the inguinal ligament to induce the 5‐min ischemic stimulus. Recording of the right superficial femoral artery was performed in the proximal third of the thigh, at least 3 cm distal from the bifurcation and above the occlusion cuff. Postdeflation recording of the superficial femoral artery was performed for 5 min. Performance of the ultrasound recordings for a single subject was performed by the same experienced sonographer.

#### Interventions

Immediately after the initial FMD assessment, a 30‐min intervention was performed. To manipulate brachial artery shear, an occlusion cuff was placed around one forearm and inflated to 0, 30, or 60 mmHg. Manipulation of shear patterns in the superficial femoral artery was performed placing the cuff around the thigh. Placement of the cuff was consistently performed around the right forearm and thigh. The order of cuff intervention (0, 30, and 60 mmHg) was randomized across the three testing days, but similar for both arteries on a testing day. Pilot observations revealed that cuff inflation to 30 and 60 mmHg acutely alters retrograde shear rate in a dose‐dependent manner, which was present in the brachial and superficial femoral artery. Brachial and superficial femoral artery mean shear rate and the pattern of shear rate (antegrade vs. retrograde) were recorded at 10‐min intervals during each intervention.

### Conduit artery diameter, blood flow, and shear rate analysis

Analysis of brachial and superficial femoral artery diameters and shear rate before, during, and after the intervention was performed using custom‐designed edge‐detection and wall‐tracking software which is largely independent of investigator bias (Woodman et al. 2001). The initial phase of image analysis involves the identification of regions of interest (ROI) on the first frame of every ultrasound study. These ROI's allow automated calibration for diameters on the B‐mode image and velocities on the Doppler strip (Black et al. 2008). Another ROI is drawn around the Doppler waveform and the peak of the waveform is automatically detected. The mean diameter measures derived from within the B‐mode diameter ROI are automatically synchronized and combined with the velocity measures derived from the Doppler ROI, at 30 Hz. Ultimately, from this synchronized diameter and velocity data, blood flow (the product of lumen cross‐sectional area and Doppler velocity [*ν*]) and shear rate (four times velocity divided by diameter) (Pyke et al. 2004); Pyke and Tschakovsky 2007) are calculated at 30 Hz. All data are written to file and retrieved for analysis in a custom‐designed analysis package (see below). We have shown that reproducibility of diameter measurements using this semiautomated software is significantly better than manual methods, reduces observer error significantly, and possesses an intraobserver, within‐day coefficient of variance (CV) of 6.7% (Woodman et al. 2001). Furthermore, our method of blood flow assessment is closely correlated with actual blood flow through a “phantom” arterial flow system (Green et al. 2002).

### Data analysis

#### FMD%

Baseline diameter, blood flow, and shear rate were calculated as the mean of data acquired across the 1 min preceding the cuff inflation period. Peak diameter following cuff deflation was automatically detected according to an algorithm which identified the maximum bracket of data (with each bracket consisting of ~100 frames), subsequent to performance of a moving window smoothing function (Black et al. 2008). FMD% was calculated as the percentage rise of this peak diameter from the preceding baseline diameter. The software described above was also used for analysis of shear rate, derived from simultaneously acquired velocity and diameter measures at 30 Hz, during the three interventions.

### Statistics

Statistical analyses were performed using SPSS 20.0 (SPSS, Chicago, IL) software. All data are reported as mean (SD) unless stated otherwise, while statistical significance was assumed at *P* < 0.05. To examine whether the impact of an increase in retrograde shear rate (“intervention”: 0, 30, vs. 60 mmHg) on endothelial function differs between arteries (“artery”: brachial vs. superficial femoral artery), we employed a linear mixed model analysis. Post hoc *t*‐tests were performed when the linear mixed model reported a significant main or interaction effect. In addition to this “traditional” approach, a recent study by Atkinson et al. (2013) indicated that inadequate scaling for FMD would be present if the upper confidence limit of the regression slope of the relationship between logarithmically transformed base diameter and peak diameter is less than 1. In such an event, FMD% may not be an appropriate measure to estimate endothelial function. We checked our data for this phenomenon, and subsequently also performed the allometric modeling solution proposed by Atkinson et al. (2013). Pearson correlations were used to examine the relation between the change in retrograde shear rate and the change FMD% induced by the intervention. FMD reproducibility was assessed by calculating the CV, using the pre‐ and postintervention retrograde shear data from the 0 mmHg cuff intervention. The CV was calculated following the classical approach based on the pooled SD, as described previously (Petrie et al. 2000). Briefly, the root mean square of the error term (square root of the error term of the adjusted mean squares) of an analysis of variance is used to calculate the pooled variance. The CV can be calculated by taking the square root of the exponent of the pooled variation minus 1, times 100 (√(exp(var) − 1) × 100).

## Results

Baseline characteristics are described in Table 1. Systolic, diastolic, and mean arterial pressures were not significantly different among the three different interventions days (*P* = 0.276, *P* = 0.783, and *P* = 0.566, respectively). At baseline, we found no difference in preintervention superficial femoral artery shear rate patterns, whereas the brachial artery showed a significantly higher baseline retrograde shear rate before the 30 mmHg intervention (Table 2). No differences in brachial artery mean or antegrade shear rate were observed between testing days (Table 2). For the brachial artery retrograde shear, the coefficient of variation is 32.4, and for the superficial femoral artery retrograde shear, the coefficient of variation is 28.0.

**Table 1.** Body characteristics in healthy subjects (*N* = 13).

Variable	Mean ± SD
Age (years)	24 ± 3
Height (cm)	185 ± 7
Weight (kg)	78.1 ± 12.1
Body mass index (kg/m^2^)	22.8 ± 2.7
Systolic blood pressure (mmHg)	119 ± 9
Diastolic blood pressure (mmHg)	74 ± 7
Mean arterial pressure (mmHg)	89 ± 7

Data are presented as mean ± SD.

**Table 2.** Preintervention resting mean, antegrade, and retrograde shear rate in the brachial and superficial femoral artery of healthy young men (*n* = 13).

	0 mmHg	30 mmHg	60 mmHg	*P*‐value
Brachial artery				
Mean shear rate (sec^−1^)	95 ± 67	59 ± 31	79 ± 52	0.109
Antegrade shear rate (sec^−1^)	108 ± 64	82 ± 30	95 ± 47	0.283
Retrograde shear rate (sec^−1^)	−13 ± 12	−24 ± 15	−16 ± 14	0.009
Superficial femoral artery				
Mean shear rate (sec^−1^)	36 ± 22	45 ± 58	23 ± 19	0.309
Antegrade shear rate (sec^−1^)	61 ± 17	75 ± 52	53 ± 15	0.191
Retrograde shear rate (sec^−1^)	−25 ± 13	−30 ± 13	−30 ± 13	0.368

Data are presented as mean ± SD. *P* values represent repeated measures ANOVA.

### Shear rate patterns

**Figure 1.**
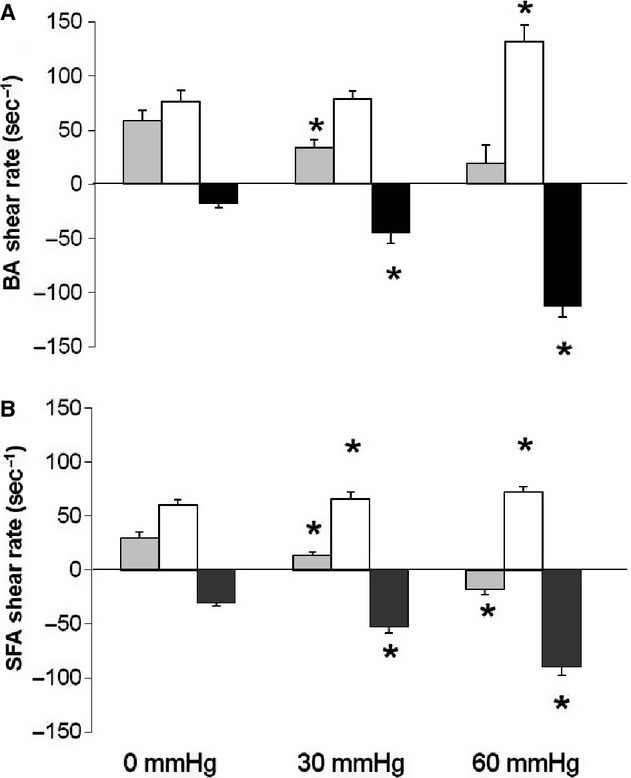
Mean (gray), antegrade (white), and retrograde shear rate (black) in the brachial (A) and superficial femoral artery (B) during the 30‐min intervention at 0, 30, and 60 mmHg in healthy young men (*n* = 13). Error bars represent SE. *Significantly different from baseline at *P* < 0.05.

Increased cuff pressure during the intervention resulted in large dose‐dependent increases in retrograde shear in both the brachial (Fig. 1A) and superficial femoral arteries (Fig. 1B). Similarly, we found that cuff inflation around the thigh resulted in a small, but significant increase in superficial femoral artery antegrade shear during the 30 and 60 mmHg interventions, leading to a small decline in mean blood flow at these cuff pressures. In the brachial artery, antegrade shear was elevated during the 60 mmHg intervention only (Fig. 1).

### Flow‐mediated vasodilation

**Figure 2.**
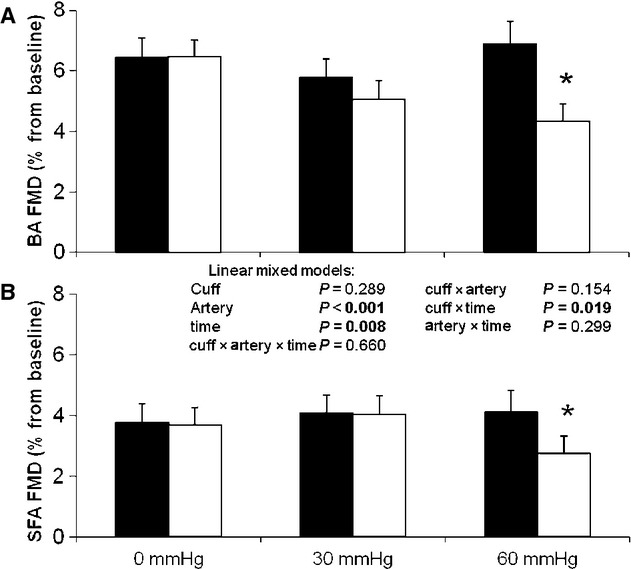
Flow‐mediated dilation before (black) and after the 30‐min intervention (white) in the brachial (A) and superficial femoral artery (B) for interventions at 0, 30, and 60 mmHg in healthy young men (*n* = 13). Error bars represent SE. *Significantly different from preintervention at *P* < 0.05.

At baseline, we found no differences in brachial artery or superficial femoral artery FMD between the three testing days, whereas the brachial artery FMD was significantly larger than the response in the superficial femoral artery (“artery”, *P* < 0.001). In both arteries, a decrease in FMD was observed as a result of cuff placement (Fig. 2; “Time × Cuff” interaction effect *P* *<* 0.05). Post hoc analysis revealed that, for both arteries, FMD did not change after the 0 mmHg and the 30 mmHg interventions, whereas a significant decrease was observed after 60 mmHg (Fig. 2). More importantly, we found that the change in FMD was not different between both arteries (Fig. 2; “Time × Cuff × Artery” interaction *P* = 0.660). A repeat of this analysis using allometric scaling, including resting diameter as a covariate, reinforced the presence of a significant impact of cuff on the change in FMD (“Time × Cuff” interaction effect: *P* < 0.05), which was not different between the brachial and superficial femoral artery (“Time × Cuff × Artery” interaction effect: *P* = 0.616). However, using the allometric scaling method, the difference in FMD between vessels was no longer present (*P* = 0.201).

**Figure 3.**
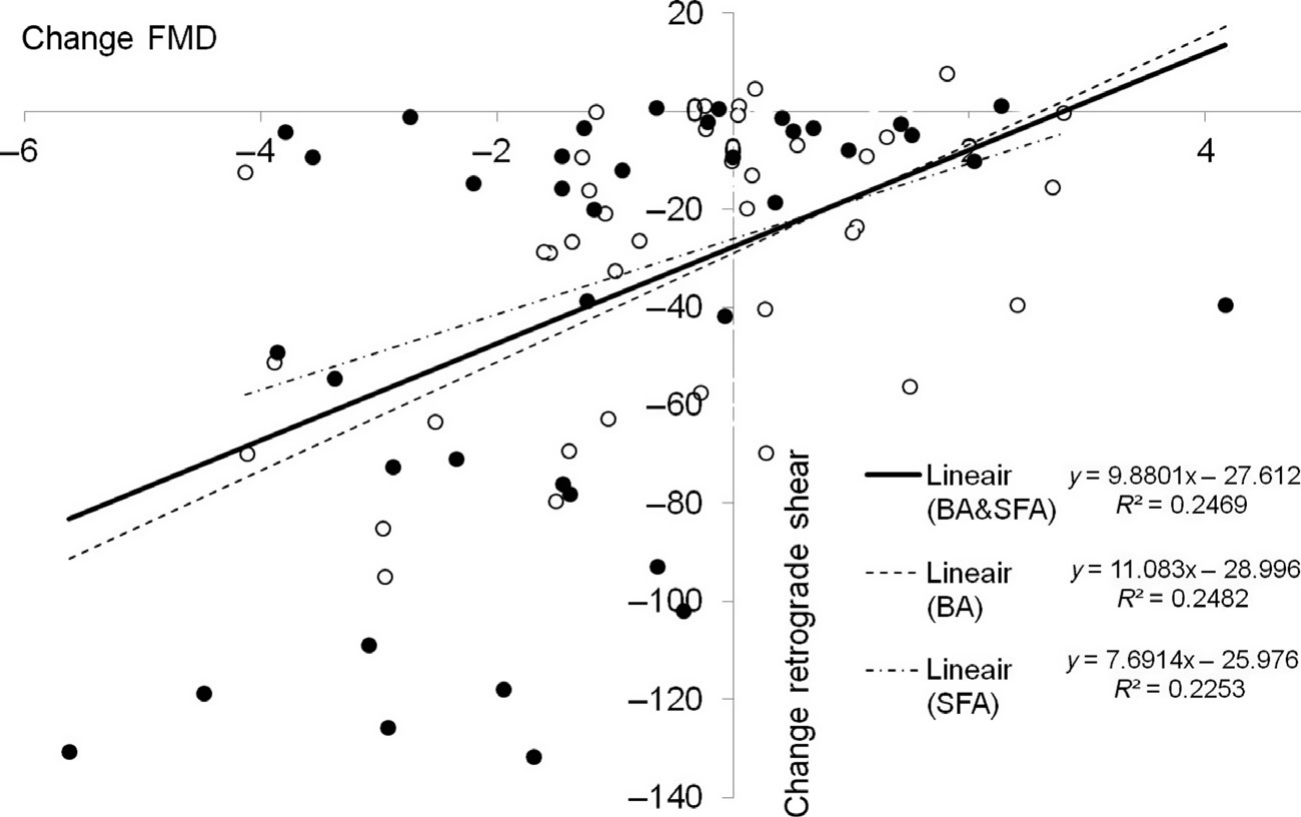
Correlation between change in retrograde shear (*y*‐axis) and change in flow‐mediated dilation (*x*‐axis) for the brachial (black dots) and superficial femoral artery (open dots).

Using the nonadjusted FMD values, we found a comparable correlation between the cuff inflation‐induced change in retrograde shear and the change in FMD in the brachial artery (*P* = 0.001, *r* = 0.498), superficial femoral artery (*P* = 0.002, *r* = 0.475), and the pooled data set (*P* < 0.001, *r* = 0.497) (Fig. 3).

For baseline diameter and shear rate area under the curve, we did not find any significant effects for any of the variables (all *P* > 0.05), except for “artery” (*P* < 0.01).

## Discussion

The purpose of this study was to examine the acute impact of dose‐dependent increases in retrograde shear on endothelial function in atherosclerotic‐prone (i.e., superficial femoral artery) and ‐resistant arteries (brachial artery) in humans. Our findings indicate that increase in retrograde shear is followed by a decrease in endothelial function, which was similarly present in an artery in the upper and lower limb. Therefore, this study provides evidence for the presence of an inverse relationship between retrograde shear and endothelial function, which is similarly present in an atherosclerosis‐prone and ‐resistant peripheral conduit artery in healthy young humans.

In line with previous findings from our laboratory (Thijssen et al. 2009), and those of others (Johnson et al. 2012); Gurovich and Braith 2013), an increase in cuff pressure resulted in an upstream, dose‐dependent increase in retrograde shear in the brachial artery. We extend this knowledge by reporting that similar dose‐dependent increases in retrograde shear are also present in the superficial femoral artery following partial cuff inflation around the thigh. This confirms that our intervention was effective, and that both brachial and superficial femoral artery retrograde shear can be manipulated using an externally placed cuff around a limb. More importantly, we examined the immediate impact of these manipulations in shear rate pattern on conduit artery endothelial function. In line with a previous observation from our laboratory, we found that an increase in retrograde shear rate decreases FMD in the brachial artery (Thijssen et al. 2009). Moreover, the superficial femoral artery responded in a similar way, indicating that short‐term exposure to retrograde shear in both atherosclerosis‐prone and atherosclerosis‐resistant arteries results in endothelial dysfunction. The similarity in responsiveness between both arteries is supported by the agreement in the slope, direction, and magnitude of correlations between the change in retrograde shear and the change in FMD in both vessels (Fig. 3).

The finding that upper and lower limb conduit arteries demonstrate a similar responsiveness to elevations in retrograde shear rate in humans in vivo contrasts with our hypothesis as well as findings from previous studies. These studies found that upper limb arteries possess a larger responsiveness when exposed to stimuli causing an increase in antegrade shear rate compared to lower limb arteries (Newcomer et al. 2004); Wray et al. 2005). One potential explanation of the different finding in our study may relate to the stimulus used. This study exposed arteries to a change in shear stress, which directly impacts endothelial function. Previous studies infused pharmacological substances or used prolonged arterial occlusions (Newcomer et al. 2004); Wray et al. 2005); forms of stimulus which depend upon distinct signal transduction pathways to those associated with shear stress, the physiologically valid approach. An alternative explanation is that we examined responsiveness to an increase in retrograde shear rate, rather than stimuli that cause an increase in antegrade shear rate.

In both arteries, the 30 mmHg intervention was not associated with a significant change in FMD, despite a significant increase in retrograde shear rate. In a previous study, Thijssen et al. (2009) found that somewhat lower cuff inflation (i.e., 25 mmHg) also caused an increase in retrograde shear rate, but did not affect brachial artery FMD. In contrast, an effect on FMD was present at 50 and 75 mmHg cuff interventions in the brachial artery (Thijssen et al. 2009). These observations between studies suggest that a “threshold” for the application of an external pressure exists that lies between 30 and 50 mmHg. Actually, the “threshold” does not relate to the pressure applied to a limb, but rather to the associated increase in retrograde shear that represents a potent stimulus to impair conduit artery endothelial function. The presence of a threshold in retrograde shear rate to impair FMD is supported by the correlation between the change in retrograde shear rate and FMD, as the regression line of both arteries crosses the *X*‐axis around −25 sec^−1^. This suggests that acute elevation in retrograde shear rate should be substantial before inducing a significant decrease in FMD, which is typically achieved by an external cuff pressure of 30–50 mmHg. It should be emphasized that the potential presence of a threshold level for retrograde shear rate is likely dependent upon the duration of the stimulus, but also possibly the vessel studied. Future studies should further examine the potential presence of a threshold for changes in FMD.

Recent studies found that changes in the contribution of NO (Padilla et al. 2011) and alpha‐adrenergic sympathetic nervous system (Casey et al. 2012) to vascular tone contribute to the magnitude of retrograde shear rate, at least in older humans. Possibly, changes in these vasoactive substances may contribute to the development of endothelial dysfunction during prolonged exposure to potentially harmful shear patterns. However, no previous study has examined the impact of prolonged exposure to elevated levels of retrograde shear rate. Future studies are warranted to examine the impact of chronic exposure to elevated levels of retrograde shear rate in humans to better understand the importance of shear pattern in the development of endothelial dysfunction.

### Clinical relevance

Previous animal and in vitro studies have demonstrated that arteries exposed to a low or oscillatory shear stress are associated with the development of atherosclerotic plaques. Although upper and lower limb arteries demonstrate differences in susceptibility for the development of atherosclerosis in humans (Dalager et al. 2007), we found that short‐term exposure to retrograde shear leads to a similar acute decrease in FMD in the both the brachial and the superficial femoral artery. The development of atherosclerosis in the superficial femoral artery may, at least in part, relate to the larger and/or longer exposure to adverse shear patterns under physiological conditions. In a previous study, Newcomer et al. (2008) found that the superficial femoral artery is exposed to lower levels of mean shear rate in the supine, seated, and standing position, possibly because of a higher retrograde shear rate. This suggests that prolonged exposure of vessels to retrograde shear rate may have detrimental effect for vascular health in humans. Elevated levels of retrograde shear have been reported in clinical populations (e.g., older humans) (Padilla et al. 2011) and can be augmented in several common clinical scenarios, such as elevations in blood pressure due to sympathetic control (Padilla et al. 2011), changes in posture (Newcomer et al. 2008), or changes in resting vascular tone, or exercise (Green et al. 2002). Therefore, the findings in this study are highly relevant from a clinical point of view.

### Limitations

Strengths of this study include the use of a tightly controlled protocol, the use of well‐trained sonographers, and the use of automated analysis software. A potential weakness is that this study does not provide insight into the mechanisms explaining the relationship between retrograde shear and endothelial function. Also, because we included a healthy group of subjects without endothelial dysfunction and/or clinical evidence of atherosclerosis, we cannot directly extrapolate our findings to other populations, including those with an increased risk for cardiovascular disease, who are likely to have developed atherosclerosis and endothelial dysfunction. Future studies should focus on these groups and examine whether vascular responses to retrograde shear rate differ between subjects with and without a priori endothelial dysfunction. Another potential limitation is that our shear stimulus, due to the possible presence of a threshold in retrograde shear as described above, is not sufficient to evoke differences in endothelial function.

In conclusion, we found that cuff inflation around the forearm and thigh resulted in a dose‐dependent increase in retrograde shear, which was comparable in both atherosclerotic‐prone and ‐resistant arteries. Moreover, we found that an increase in retrograde shear is associated with a decrease in conduit artery endothelial function, while this relation between retrograde shear and endothelial function was similar between the brachial and superficial femoral arteries. This demonstrates, for the first time in humans, that the potentially detrimental impact of short‐term increases in retrograde shear is associated with a similar decrease in endothelial function in atherosclerosis‐prone and ‐resistant vessels.

## Acknowledgments

We would like to thank Chris Reed for his assistance with software development. We would also like to thank Erik Stroeken and Klaas‐Jan Duplant for their assistance.

## Conflict of Interest

None declared.

## References

[b1] Atkinson G. Batterham A. M. Thijssen D. H. Green D. J. 2013 A new approach to improve the specificity of flow‐mediated dilation for indicating endothelial function in cardiovascular research. J. Hypertens.; 31:287-291 23169234 10.1097/HJH.0b013e32835b8164

[b2] Black M. A. Cable N. T. Thijssen D. H. Green D. J. 2008 Importance of measuring the time course of flow‐mediated dilatation in humans. Hypertension; 51:203-210 18086954 10.1161/HYPERTENSIONAHA.107.101014

[b3] Casey D. P. Padilla J. Joyner M. J. 2012 Alpha‐adrenergic vasoconstriction contributes to the age‐related increase in conduit artery retrograde and oscillatory shear. Hypertension; 60:1016-1022 22949528 10.1161/HYPERTENSIONAHA.112.200618 PMC3483145

[b4] Chappell D. C. Varner S. E. Nerem R. M. Medford R. M. Alexander R. W. 1998 Oscillatory shear stress stimulates adhesion molecule expression in cultured human endothelium. Circ. Res.; 82:532-539 9529157 10.1161/01.res.82.5.532

[b5] Corretti M. C. Anderson T. J. Benjamin E. J. Celermajer D. Charbonneau F. Creager M. A. 2002 Guidelines for the ultrasound assessment of endothelial‐dependent flow‐mediated vasodilation of the brachial artery: a report of the International Brachial Artery Reactivity Task Force. J. Am. Coll. Cardiol.; 39:257-265 11788217 10.1016/s0735-1097(01)01746-6

[b6] Dalager S. Paaske W. P. Kristensen I. B. Laurberg J. M. Falk E. 2007 Artery‐related differences in atherosclerosis expression: implications for atherogenesis and dynamics in intima‐media thickness. Stroke; 38:2698-2705 17761918 10.1161/STROKEAHA.107.486480

[b7] Davignon J. Ganz P. 2004 Role of endothelial dysfunction in atherosclerosis. Circulation; 109:III27-III32 15198963 10.1161/01.CIR.0000131515.03336.f8

[b8] Green D. Cheetham C. Reed C. Dembo L. O'Driscoll G. 2002 Assessment of brachial artery blood flow across the cardiac cycle: retrograde flows during cycle ergometry. J. Appl. Physiol.; 93:361-368 12070226 10.1152/japplphysiol.00051.2002

[b9] Green D. J. Bilsborough W. Naylor L. H. Reed C. Wright J. O'Driscoll G. 2005 Comparison of forearm blood flow responses to incremental handgrip and cycle ergometer exercise: relative contribution of nitric oxide. J. Physiol.; 562:617-628 15513940 10.1113/jphysiol.2004.075929 PMC1665516

[b10] Gurovich A. N. Braith R. W. 2013 Enhanced external counterpulsation creates acute blood flow patterns responsible for improved flow‐mediated dilation in humans. Hypertens. Res.; 36:297-305 23076403 10.1038/hr.2012.169

[b11] Hsiai T. K. Cho S. K. Wong P. K. Ing M. Salazar A. Sevanian A. 2003 Monocyte recruitment to endothelial cells in response to oscillatory shear stress. FASEB J.; 17:1648-1657 12958171 10.1096/fj.02-1064com PMC4108745

[b12] Johnson B. D. Padilla J. Wallace J. P. 2012 The exercise dose affects oxidative stress and brachial artery flow‐mediated dilation in trained men. Eur. J. Appl. Physiol.; 122:33-42 21472439 10.1007/s00421-011-1946-8

[b13] Kooijman M. Thijssen D. H. de Groot P. C. Bleeker M. W. van Kuppevelt H. J. Green D. J. 2008 Flow‐mediated dilatation in the superficial femoral artery is nitric oxide mediated in humans. J. Physiol.; 586:1137-1145 18096601 10.1113/jphysiol.2007.145722 PMC2375653

[b15] Mullen M. J. Kharbanda R. K. Cross J. Donald A. E. Taylor M. Vallance P. 2001 Heterogenous nature of flow‐mediated dilatation in human conduit arteries in vivo: relevance to endothelial dysfunction in hypercholesterolemia. Circ. Res.; 88:145-151 11157665 10.1161/01.res.88.2.145

[b16] Newcomer S. C. Leuenberger U. A. Hogeman C. S. Handly B. D. Proctor D. N. 2004 Different vasodilator responses of human arms and legs. J. Physiol.; 556:1001-1011 14990681 10.1113/jphysiol.2003.059717 PMC1665001

[b17] Newcomer S. C. Sauder C. L. Kuipers N. T. Laughlin M. H. Ray C. A. 2008 Effects of posture on shear rates in human brachial and superficial femoral arteries. Am. J. Physiol. Heart Circ. Physiol.; 294:H1833-H1839 18245564 10.1152/ajpheart.01108.2007 PMC3289057

[b18] Newcomer S. C. Thijssen D. H. Green D. J. 2011 Effects of exercise on endothelium and endothelium/smooth muscle cross talk: role of exercise‐induced hemodynamics. J. Appl. Physiol.; 111:311-320 21436465 10.1152/japplphysiol.00033.2011

[b19] Niebauer J. Cooke J. P. 1996 Cardiovascular effects of exercise: role of endothelial shear stress. J. Am. Coll. Cardiol.; 28:1652-1660 8962548 10.1016/S0735-1097(96)00393-2

[b20] Nishiyama S. K. Walter Wray D. Berkstresser K. Ramaswamy M. Richardson R. S. 2007 Limb‐specific differences in flow‐mediated dilation: the role of shear rate. J. Appl. Physiol.; 103:843-851 17556495 10.1152/japplphysiol.00273.2007

[b21] Padilla J. Simmons G. H. Fadel P. J. Laughlin M. H. Joyner M. J. Casey D. P. 2011 Impact of aging on conduit artery retrograde and oscillatory shear at rest and during exercise: role of nitric oxide. Hypertension; 57:484-489 21263118 10.1161/HYPERTENSIONAHA.110.165365 PMC3049300

[b22] Parker B. A. Ridout S. J. Proctor D. N. 2006 Age and flow‐mediated dilation: a comparison of dilatory responsiveness in the brachial and popliteal arteries. Am. J. Physiol. Heart Circ. Physiol.; 291:H3043-H3049 16861699 10.1152/ajpheart.00190.2006

[b23] Petrie J. R. Perry C. Cleland S. J. Murray L. S. Elliott H. L. Connell J. M. 2000 Forearm plethysmography: does the right arm know what the left is doing? Clin. Sci. (Lond.); 98:209-210 10657277

[b24] Pyke K. E. Tschakovsky M. E. 2007 Peak vs. total reactive hyperemia: which determines the magnitude of flow‐mediated dilation? J. Appl. Physiol.; 102:1510-1519 17170205 10.1152/japplphysiol.01024.2006

[b25] Pyke K. E. Dwyer E. M. Tschakovsky M. E. 2004 Impact of controlling shear rate on flow‐mediated dilation responses in the brachial artery of humans. J. Appl. Physiol.; 97:499-508 15064302 10.1152/japplphysiol.01245.2003

[b26] Thijssen D. H. Dawson E. A. Tinken T. M. Cable N. T. Green D. J. 2009 Retrograde flow and shear rate acutely impair endothelial function in humans. Hypertension; 53:986-992 19380611 10.1161/HYPERTENSIONAHA.109.131508

[b27] Thijssen D. H. Black M. A. Pyke K. E. Padilla J. Atkinson G. Harris R. A. 2011 Assessment of flow‐mediated dilation in humans: a methodological and physiological guideline. Am. J. Physiol. Heart Circ. Physiol.; 300:H2-H12 20952670 10.1152/ajpheart.00471.2010 PMC3023245

[b28] Tinken T. M. Thijssen D. H. Hopkins N. Black M. A. Dawson E. A. Minson C. T. 2009 Impact of shear rate modulation on vascular function in humans. Hypertension; 54:278-285 19546374 10.1161/HYPERTENSIONAHA.109.134361 PMC3012006

[b29] Tinken T. M. Thijssen D. H. Hopkins N. Dawson E. A. Cable N. T. Green D. J. 2010 Shear stress mediates endothelial adaptations to exercise training in humans. Hypertension; 55:312-318 20048193 10.1161/HYPERTENSIONAHA.109.146282

[b30] Wang C. Baker B. M. Chen C. S. Schwartz M. A. 2013 Endothelial cell sensing of flow direction. Arterioscler. Thromb. Vasc. Biol.; 33:2130-2136 23814115 10.1161/ATVBAHA.113.301826 PMC3812824

[b31] Widlansky M. E. Gokce N. Keaney J. F. Jr. Vita J. A. 2003 The clinical implications of endothelial dysfunction. J. Am. Coll. Cardiol.; 42:1149-1160 14522472 10.1016/s0735-1097(03)00994-x

[b32] Woodman R. J. Playford D. A. Watts G. F. Cheetham C. Reed C. Taylor R. R. 2001 Improved analysis of brachial artery ultrasound using a novel edge‐detection software system. J. Appl. Physiol.; 91:929-937 11457812 10.1152/jappl.2001.91.2.929

[b33] Wray D. W. Uberoi A. Lawrenson L. Richardson R. S. 2005 Heterogeneous limb vascular responsiveness to shear stimuli during dynamic exercise in humans. J. Appl. Physiol.; 99:81-86 15718401 10.1152/japplphysiol.01285.2004

